# Vergleichende Bewertung der spongiösen Knochendichte in Hounsfield-Einheiten im lumbalen nativen CT-Schnittbild zur Osteoporosediagnostik und Frakturrisikobestimmung durch verschiedene Untersucher

**DOI:** 10.1007/s00132-024-04587-3

**Published:** 2024-12-03

**Authors:** Julian Ramin Andresen, Guido Schröder, Thomas Haider, Christoph Kopetsch, Claus Maximilian Kullen, Hans Christof Schober, Reimer Andresen

**Affiliations:** 1 Klinische Abteilung für Unfallchirurgie, Universitätsklinik für Orthopädie und Unfallchirurgie, Medizinische Universität Wien, Währinger Gürtel 18–20, 1090 Wien, Österreich; 2 Klinik für Unfall‑, Hand- und Wiederherstellungschirurgie, Universitätsmedizin Rostock, Rostock, Deutschland; 3 Institut für Diagnostische und Interventionelle Radiologie/Neuroradiologie, Westküstenklinikum Heide, Akademisches Lehrkrankenhaus der Universitäten Kiel, Lübeck und Hamburg, Heide, Heide, Deutschland; 4 OrthoCoast, Praxis für Orthopädie und Osteologie, Wolgast, Deutschland

**Keywords:** Knochenverlust, altersbedingter, Knochenmineralgehalt, Insuffizienzfrakturen, Lumbosakralregion, Osteoporotische Fraktur, Bone loss, age-related, Bone mineral density, Insufficiency fractures, Lumbosacral region, Osteoporotic fracture

## Abstract

**Hintergrund:**

Ein zunehmender Verlust an Knochenmineralgehalt (KMG) am Achsenskelett führt zu einer Osteoporose und Frakturen, wobei sich thorakal und thorakolumbal eine Häufung findet.

**Fragestellung:**

Inwieweit eine untersucherunabhängige Abschätzung zum Ausmaß einer Osteoporose und Frakturrisikobestimmung mittels spongiöser Dichtebestimmung in Hounsfield-Einheiten (HU) in der Wirbelsäule möglich ist, sollte überprüft werden. Lassen sich aus den HU-Werten quantitative KMG-Werte berechnen, war die nächste Frage.

**PatientInnen und Methode:**

Es wurden 225 PatientInnen (Pat.) mit einem Durchschnittsalter von 64,9 ± 13,1 Jahren und einem Body-Mass-Index (BMI) von 26,8 ± 6,8 kg/m^2^, hiervon 37 Männer und 188 Frauen, mit der Frage nach dem Vorhandensein einer Osteoporose untersucht. Eine Bestimmung des KMG in mg/cm^3^ erfolgte mittels quantitativer Computertomographie (QCT) im lumbalen Bereich. Es erfolgte nach Anonymisierung durch drei erfahrene Radiologen eine zusätzliche Messung der Spongiosadichte in HU in denselben Wirbelköpern (insgesamt 675 Wirbelkörper), jeweils durch eine im mittvertebralen spongiösen Raum positionierte „region of interest“ (ROI) im sagittal reformierten CT-Schnittbild. In zusätzlich durchgeführten lateralen Röntgenaufnahmen der Brust- (BWS) und Lendenwirbelsäule (LWS) erfolgte die Detektion und Gradeinteilung von Wirbelkörperfrakturen. Zur gleichen Zeit aufgetretene Sakruminsuffizienzfrakturen wurden miterfasst.

**Ergebnisse:**

Der mediane KMG betrug 73,2 (57,05–104,17) mg/cm^3^ und der mediane HU 89,93 (67,90–126,95). Bei einer Korrelation von 0,988 (*p* < 0,001) lassen sich nach der Formel: *Xq* *=* *12,1* *+* *0,68* *×* *HU* quantitative Werte in mg/cm^3^ errechnen. Bei HU-Werten < 69,84 und einem KMG der LWS unterhalb von 59,54 mg/cm^3^ fanden sich signifikant vermehrte OWF. Bei 137/225 Pat. fanden sich mindestens eine OWF. Bei 17/137 Pat. fanden sich zusätzlich Sakrumfrakturen, diese Pat. zeigten mit einem medianen KMG von 41,81 (16,2–53,7) mg/cm^3^ die signifikant niedrigsten Werte. Unabhängig von den Untersuchern wurden vergleichbare HU-Werte bestimmt (*p* > 0,05).

**Diskussion:**

Die spongiösen Dichtemessungen in HU-Werten lassen sich in quantitative KMG-Werte in mg/cm^3^ umrechnen, womit eine gute Abschätzung einer Osteoporose und Frakturrisikobestimmung möglich wird. Unter Berücksichtigung der gewonnenen Ergebnisse erscheint eine opportunistische Auswertung allein mittels HU-Werten im nativen CT gut möglich. Hierbei kommen erfahrene Untersucher zu vergleichbaren Ergebnissen.

**Graphic abstract:**

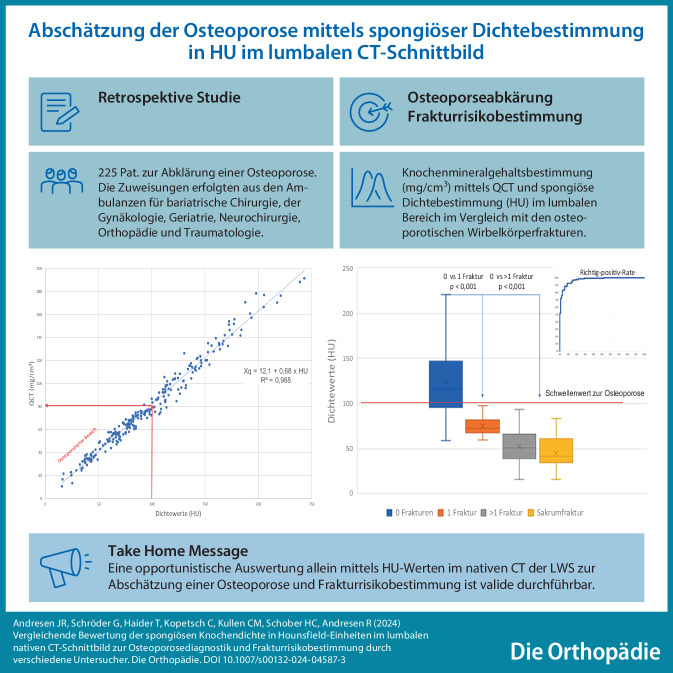

## Abstract (en)

**Background:**

An increasing loss of bone mineral density (BMD) in the axial skeleton leads to osteoporosis and fractures, with an increase found in the thoracic and thoracolumbar regions.

**Research question:**

The extent to which an examiner-independent assessment of the extent of osteoporosis and fracture risk determination is possible by determining the trabecular density in Hounsfield units (HU) in the spine should be examined. The next question was whether quantitative BMD values can be calculated from the HU values.

**Patients and methods:**

225 patients (pt.) with an average age of 64.9 ± 13.1 years and a body-mass-index (BMI) of 26.8 ± 6.8 kg/m^2^, of which 37 were men and 188 were women, were examined to determine whether they had osteoporosis. The BMD was determined in mg/cm^3^ using quantitative computed tomography (QCT) in the lumbar region. After anonymization by three experienced radiologists, an additional measurement of the trabecular bone density in HU, was carried out in the same vertebral bodies (a total of 675 vertebral bodies), each using a region of interest (ROI) positioned in the midvertebral cancellous space in the sagittal reformed CT image. In additional lateral X‑rays of the thoracic and lumbar spine, vertebral fractures were detected and graded. Sacral insufficiency fractures that occurred at the same time were also recorded.

**Results:**

The median BMD was 73.2 (57.05–104.17) mg/cm^3^ and the median HU was 89.93 (67.90–126.95). With a correlation of 0.988 (*p* < 0.001), quantitative values in mg/cm^3^ can be calculated using the following formula: *Xq* *=* *12.1* *+* *0.68* *×* *HU*. With HU values less than 69.84 and a BMD of the lumbar spine below 59.54 mg/cm^3^, there was a significantly increased number of OVF. At least one OVF was found in 137/225 pt. In 17/137 pt., sacral fractures were also found; these patients showed the significantly lowest values with a median BMD of 41.81 (16.2–53.7) mg/cm^3^. Comparable HU values were determined independently of the examiners (*p* > 0.05).

**Discussion:**

The trabecular density measurements in HU values can be converted into quantitative BMD values in mg/cm^3^, which enables a good assessment of osteoporosis and fracture risk. Taking the results obtained into account, an opportunistic evaluation using HU values in native CT alone seems quite possible. Experienced examiners have arrived at comparable results.

Osteoporotische Wirbelkörperfraktur (OWF) laufen häufig stumm ab und werden somit gar nicht oder erst spät erkannt. Eine antiosteoporotische medikamentöse Therapie zur Frakturrisikominimierung findet somit nicht oder erst verspätet statt. Aus unterschiedlicher medizinischer Indikation liegen häufig CT-Untersuchungen vor, hier könnte die Bestimmung der trabekulären Wirbelkörperdichte in Hounsfield-Einheiten (HU) eine Abschätzung einer vorliegenden Osteopenie/Osteoporose ohne zusätzliche Strahlenbelastung ermöglichen. In der nachfolgenden Studie konnte ein Schwellenwert für Osteopenie von < 160 HU und für Osteoporose von < 100 HU in nativen CT-Untersuchen der LWS bestimmt werden.

## Einleitung

Ein zunehmender Verlust an Knochenmineralgehalt (KMG) im Achsenskelett führt zu Osteoporose und vermehrten osteoporotischen Wirbelkörperfrakturen (OWF) [1]. Jedes Jahr gibt es weltweit über 1,4 Mio. Fälle von klinisch auffälligen OWF [16]. In Bezug auf die Epidemiologie ist die OWF die häufigste osteoporotische Fraktur, die bei 30–50 % der Bevölkerung über 50 Jahren auftritt [36]. Pat., die eine OWF erlitten haben, haben ein bis zu 5‑mal erhöhtes Risiko, innerhalb des nächsten Jahres einen weiteren Wirbelbruch zu erleiden [21]. Langfristig führen diese Frakturen zu einer Minderung der Lebensqualität und einer erhöhten Mortalität [25, 35].

Zu den Risikofaktoren für OWF gehören insbesondere frühere Fragilitätsfrakturen, hohes Alter, weibliches Geschlecht, Vitamin-D-Mangel, rheumatoide Arthritis, Behandlung mit Kortikosteroiden, Immobilisation, niedriger Body-Mass-Index (BMI), exzessiver Alkoholkonsum, Nikotinabusus und weitere sekundäre Ursachen für Osteoporose [12, 17, 19, 21, 22, 25, 26, 29, 35, 37, 42]. Die Frakturverteilung im Achsenskelett zeigt einen Häufigkeitsgipfel im mittleren thorakalen und thorakolumbalen Bereich [3, 23, 31], wobei den Krümmungen der Wirbelsäule in der Sagittalebene eine hohe biomechanische Bedeutung zukommt [7].

Auch bei manifester Osteoporose werden Insuffizienzfrakturen im zervikalen Abschnitt aufgrund ihrer besonderen Mikroarchitektur und relativ hohen Dichte nur selten gefunden; ein Frakturschwellenwert wird hier scheinbar nicht unterschritten [31]. Mit zunehmender Schwere der Osteoporose treten jedoch vermehrt Insuffizienzfrakturen im sakralen Abschnitt auf [2].

Als Goldstandard für die Bestimmung der Knochendichte und Identifizierung einer Osteoporose gilt die Dual-Energie-Röntgenabsorptiometrie (DEXA) [10], wobei ein T‑Score von −1,5 bis −2,5 als Osteopenie und niedriger als −2,5 als Osteoporose definiert wird [9]. Als Alternative ist auch eine quantitative Computertomographie (QCT) zur Bestimmung des spongiösen KMG im lumbalen Wirbelsäulenabschnitt möglich, hier wird ein KMG < 120 mg/cm^3^ als Osteopenie und < 80 mg/cm^3^ als Osteoporose definiert [11, 13].

Inwieweit eine untersucherunabhängige Abschätzung zum Ausmaß einer Demineralisation/Schwere einer Osteoporose und Frakturrisikobestimmung mittels spongiöser Dichtebestimmung in Hounsfield-Units (HU) in der Wirbelsäule möglich ist, sollte überprüft werden. Lassen sich aus den HU-Werten quantitative KMG-Werte berechnen war die nächste Frage.

## PatientInnen und Methode

### Studiendesign und Ethikvotum

Für die Durchführung der multizentrischen, retrospektiven Studie liegt ein positives Ethikvotum der zuständigen Universitätsmedizin (AZ: D 471/24) vor.

### Patientengut

Es wurden 225 PatientInnen (Pat.) mit einem Durchschnittsalter (DSA) von 64,9 ± 13,1 Jahren und einem Body-Mass-Index (BMI) von 26,8 ± 6,8 kg/m^2^, hiervon 37 Männer mit einem DSA von 60,0 ± 14,3 Jahren und einem BMI von 28,4 ± 5,7 kg/m^2^ und 188 Frauen mit einem DSA von 65,8 ± 12,7 Jahren und einem BMI von 26,5 ± 7,0 kg/m^2^, mit der Frage nach dem Vorhandensein einer Osteoporose untersucht. Die Zuweisungen erfolgten aus den Ambulanzen für bariatrische Chirurgie, der Gynäkologie, Geriatrie, Neurochirurgie, Orthopädie und Traumatologie.

### Diagnostik

**Abb. 1:**
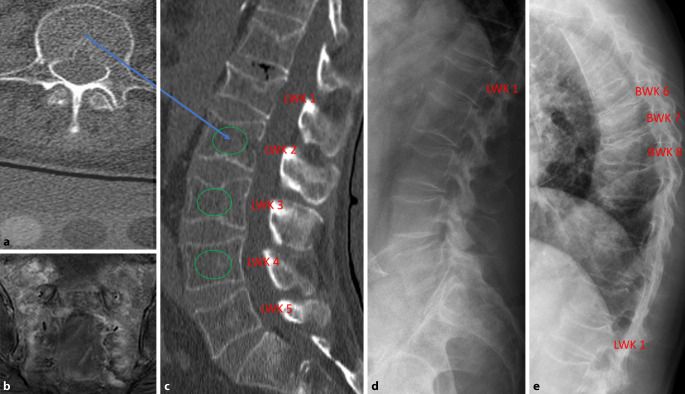
**a** Mittvertebrales axiales CT-Schnittbild mit darunterliegendem Referenzkörper für die lumbale Osteodensitometrie. **b** Koronares MRT-Schnittbild (STIR-Sequenz) mit Darstellung einer beidseitigen frischen Sakrumfraktur. **c** Sagittal reformiertes CT-Schnittbild mit eingezeichneter ellipsoider „region of interest“ in die nicht frakturierten Lendenwirbelkörper LWK 2, LWK 3 und LWK 4 (*grüne Kreise*). **d** und **e** laterale Röntgenaufnahme der Lendenwirbelsäule und Brustwirbelsäule zur Detektion der osteoporotischen Wirbelkörperfraktur. Bei der 78-jährigen Pat. ergab die lumbale Bestimmung mittels quantitativer Computertomographie einen Knochenmineralgehalt (KMG) von 30,8 mg/cm^3^ als Mittelwert aus LWK 2, LWK 3 und LWK 4, dieses ist Ausdruck einer schweren Demineralisation. Die spongiöse Dichtemessung ergab einen Mittelwert von 27,8 HU und nach Umrechnung ein KMG von 31 mg/cm^3^, welches sehr gut mit der gemessenen Knochendichte korrespondiert; *BWK* Brustwirbelkörper

Eine Bestimmung des KMG in mg/cm^3^ erfolgte mittels QCT (GE-Revolution EVO/64 Zeilen CT sowie Mindways Software 3D Volumetric QCT Spine, Austin, Tx, USA) in Höhe von LWK 1, LWK 2 und LWK 3. Es erfolgte nach Anonymisierung durch drei erfahrene Radiologen, mit mindestens 10 Jahren Erfahrung in der muskuloskelettalen Bildgebung, eine zusätzliche Messung der Spongiosadichte in HU in denselben Wirbelköpern (insgesamt 675 Wirbelkörper), jeweils durch eine im mittvertebralen spongiösen Raum manuell positionierte ellipsoide ROI im sagittal reformierten CT-Schnittbild, bei einer Schichtdicke von 2 mm und einer Fenstereinstellung von C = 400/W = 1600. In zusätzlich durchgeführten lateralen Röntgenaufnahmen der BWS und LWS erfolgte die Detektion und Gradeinteilung von Wirbelkörperfrakturen nach Genant et al. [14]. Dabei wird das Ausmaß des Höhenverlusts der Wirbel berücksichtigt, bei dem ein leichter Schweregrad eine vordere, mittlere oder hintere Höhenminderung von 20–25 % (G1), ein mäßiger Schweregrad von 25–40 % (G2) und ein schwerer Schweregrad von > 40 % (G3) im Vergleich zu den angrenzenden Wirbeln aufweist. Bei klinischem Verdacht auf Insuffizienzfrakturen im Sakrum wurden zur gleichen Zeit durchgeführte MRT-Untersuchungen mit ausgewertet. (Abb. 1).

### Statistik

Die erhobenen Daten wurden mit dem statistischen Softwarepaket SPSS, Version 23.0 (SPSS Inc., Armonk, NY, USA) analysiert. Die Beschreibung der quantitativen Merkmale erfolgte bei parametrischen Tests jeweils als Mittelwert (M), Standardabweichung (SD) und Anzahl (*n*) der verfügbaren Beobachtungen, sie wurden mithilfe des Intervalls Mittelwert ± Standardabweichung (M ± SD) dargestellt. Bei nichtparametrischen Tests erfolgte die Darstellung jeweils als Median mit erstem und drittem Quartil (Q1–Q3). Um eine Korrelation zwischen 2 Variablen zu beschreiben, wurde der Spearman-Korrelationskoeffizient berechnet (r). Mittels Regressionsanalyse wurde der QCT-Wert (Xq) unter Verwendung einer verallgemeinerten Schätzungsgleichung bestimmt. Alle *p*-Werte sind das Resultat zweiseitiger statistischer Tests: prinzipiell wird *p* < 0,05 als signifikant angesehen. Gleichzeitig wurden die Effektstärken nach Cohen berechnet und Werte < 0,5 als kleiner, zwischen 0,5 und 0,8 als mittlerer sowie > 0,8 als großer Effekt angenommen. Für Vergleiche zwischen den verschiedenen Untersuchern kam, in Abhängigkeit vom Resultat des Shapiro-Wilk-Tests auf Normalverteilung, der Kruskal-Wallis-Test zum Einsatz. Für den Vergleich der Pat.-Gruppe ohne, mit einer, mit mehr als einer Fraktur sowie mit zusätzlich vorhandenen Sakrumfrakturen wurde der ANOVA-Test herangezogen. Mittels einer ROC-Analyse wurde die Vorhersagekraft hinsichtlich Frakturrisikoabschätzung unter Berücksichtigung der HU- und QCT durchgeführt.

## Ergebnisse

**Abb. 2:**
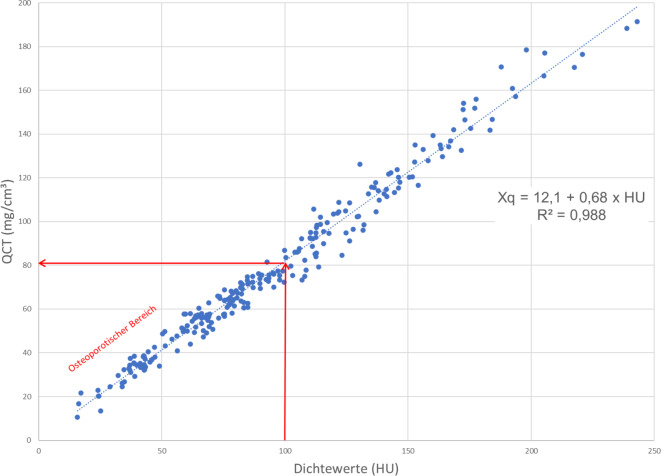
Zusammenhang der spongiösen Dichtewerte in HU mit den lumbalen QCT(quantitative Computertomographie)-Werten in mg/cm^3^. Bei 100 HU ergibt sich ein Knochenmineralgehalt von 80,1 mg/cm^3^, diese Dichtewerte entsprechen dem jeweils festgelegten quantitativen Schwellenwert zur Osteoporose [9, 11]

**Abb. 3:**
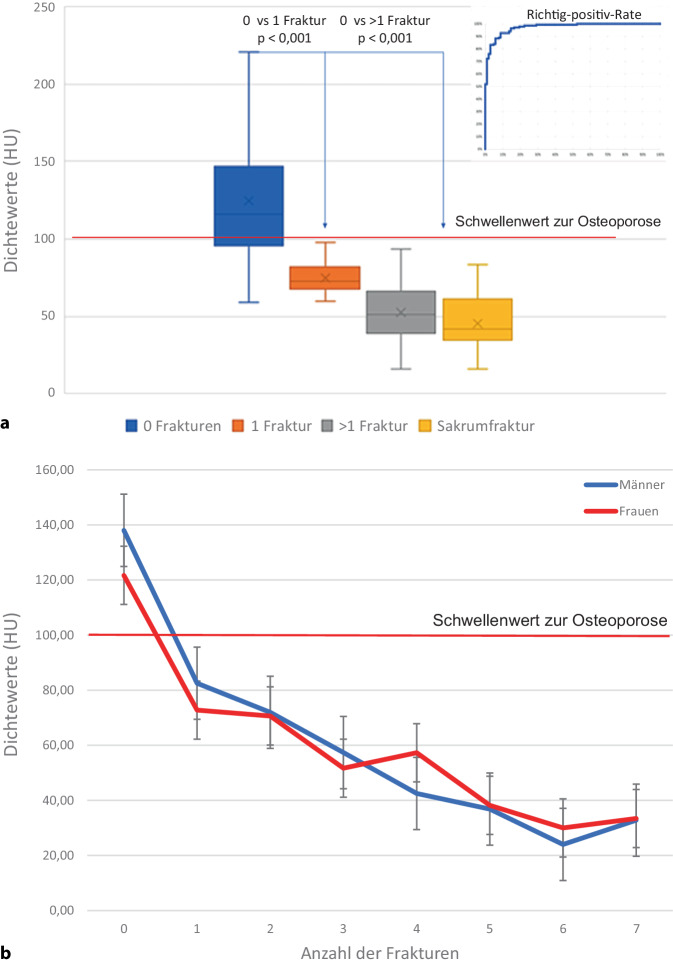
**a** Die Mittelwerte der Dichtemessung in HU zeigen einen signifikanten Unterschied (*p* < 0,001) zwischen Pat. ohne und mit mindestens einer osteoporotischen Wirbelkörperfraktur (OWF). Pat. mit Sakrumfrakturen haben die niedrigste Knochendichte, unterscheiden sich jedoch nicht signifikant von Pat. mit mehr als einer Fraktur entlang der Wirbelsäule. Oberhalb des Osteoporoseschwellenwertes von 100 HU finden sich keine Frakturen. Die Aussagekraft für die Frakturrisikobestimmung ist mit 93 % in der Richtig-positiv-Rate sehr hoch. **b** Zusammenhang zwischen den spongiösen lumbalen Dichtewerten in HU und der Anzahl von OWF am Achsenskelett. Oberhalb des Osteoporoseschwellenwertes von 100 HU finden sich keine OWF

Der mediane KMG betrug 73,2 (57,05–104,17) mg/cm^3^ (Tab. 1) und der mediane HU 89,93 (67,90–126,95), (Tab. 2). Bei einer Korrelation von R^2^ = 0,988 (*p* < 0,001), (Tab. 3) lassen sich nach folgender Formel: *Xq* *=* *12,1* *+* *0,68* *×* *HU* quantitative Werte in mg/cm^3^ errechnen. Für 100 HU ergibt sich ein KMG von 80,1 mg/cm^3^, für 160 HU ein KMG von 120,9 mg/cm^3^ (Abb. 2). Oberhalb von 100 HU fanden sich keine OWF. Bei HU-Werten kleiner 69,84 und einem KMG der LWS unterhalb von 59,54 mg/cm^3^ fanden sich, bei einer Effektstärke von 0,89, signifikant vermehrte Sinterungsfrakturen im mittleren thorakalen, thorakolumbalen und sakralen Bereich. Bei 137/225 Pat. fand sich mindestens eine Sinterungsfraktur, wobei kranial von BWK 5 keine Fraktur detektiert wurde. Bei 17/137 Pat. fanden sich zusätzlich Sakrumfrakturen, diese Pat. zeigten mit einem medianen KMG von 41,81 (16,2–53,7) mg/cm^3^ die signifikant niedrigsten Werte (Abb. 3a und b), unterschieden sich jedoch nicht signifikant von den Pat. mit mehr als einer OWF. Die Richtig-positiv-Rate zur Vorhersage der OWF beträgt 93 % (Abb. 3b). Eine Pat.-Übersicht und Charakterisierung der Gesamtstichprobe ist in Tab. 4 wiedergegeben.

**Tab. 1:** QCT-Werte (mg/cm^3^) der Lendenwirbel 1 bis 3

	*n*	Median (Q1–Q3)
Alle Lendenwirbel (L1–L3)	675	73,2 (57,05–104,17)

**Tab. 2:** Hounsfield-Einheiten (HU) der Lendenwirbel 1 bis 3

	*n*	Median (Q1–Q3)
Alle Lendenwirbel (L1–L3)	675	89,93 (67,90–126,95)

**Tab. 3:** Korrelation zwischen den HU- und den QCT-Mittelwerten mit dem entsprechenden Korrelationskoeffizienten nach Pearson®

	*n*	Pearson’s Korrelation (r)	*P*-Wert
Alle Lendenwirbel (L1–L3)	675	0,988	< 0,001

**Tab. 4:** Pat.-Beschreibung nach Geschlecht, Alter, BMI und Anzahl der OWF

	Pat. (*n* = 225)	Männer (*n* = 37)	Frauen (*n* = 188)
Alter (in Jahren)	64,9 ± 13,1	60,0 ± 14,3	65,8 ± 12,7
BMI (kg/m^2^)	26,8 ± 6,8	28,4 ± 5,7	26,5 ± 7,0
Pat. mit mindestens einer Fraktur (*n*)	137	27	110
Durchschnittliche OWF-Häufung pro Pat.	1,57	1,07	1,69
Betroffene Wirbelkörper (*n*) mit Grad der Deformität nach Genant et al. ([14]; Abb. 4)	215	29	186
T5	6 (4 × G1, 2 × G2)	0	6 (4 × G1, 2 × G2)
T6	9 (6 × G1, 3 × G2)	0	9 (6 × G1, 3 × G2)
T7	27 (19 × G1, 7 × G2, 1 × G3)	2 (1 × G1, 1 × G2)	25 (18 × G1, 6 × G2, 1 × G3)
T8	19 (12 × G1, 7 × G2)	2 (1 × G1, 1 × G2)	17 (11 × G1, 6 × G2)
T9	14 (9 × G1, 4 × G2, 1 × G3)	2 (1 × G1, 1 × G2)	12 (8 × G1, 3 × G2, 1 × G3)
T10	8 (2 × G1, 5 × G2, 1 × G3)	1 (G2)	7 (2 × G1, 4 × G2, 1 × G3)
T11	14 (3 × G1, 7 × G2, 4 × G3)	3 (1 × G1, 1 × G2, 1 × G3)	11 (2 × G1, 6 × G2, 3 × G3)
T12	26 (12 × G1, 9 × G2, 5 × G3)	3 (2 × G1, 1 × G2)	23 (10 × G1, 8 × G2, 5 × G3)
L1	37 (7 × G1, 25 × G2, 5 × G3)	4 (2 × G1, 2 × G2)	33 (5 × G1, 23 × G2, 5 × G3)
L2	17 (7 × G1, 10 × G2)	2 (1 × G1, 1 × G2)	15 (6 × G1, 9 × G2)
L3	19 (10 × G1, 8 × G2, 1 × G3)	6 (4 × G1, 2 × G2)	13 (6 × G1, 6 × G2, 1 × G3)
L4	16 (8 × G1, 7 × G2, 1 × G3)	2 (1 × G1, 1 × G2)	14 (7 × G1, 6 × G2, 1 × G3)
L5	3 (1 × G1, 2 × G2)	2 (1 × G1, 1 × G2)	1 (G2)
Sakrumfrakturen einseitig beidseitig	17 1 16	1 – 1	16 1 15

*BMI* Body-Mass-Index, *OWF* osteoporotische Wirbelkörperfraktur

**Abb. 4:**
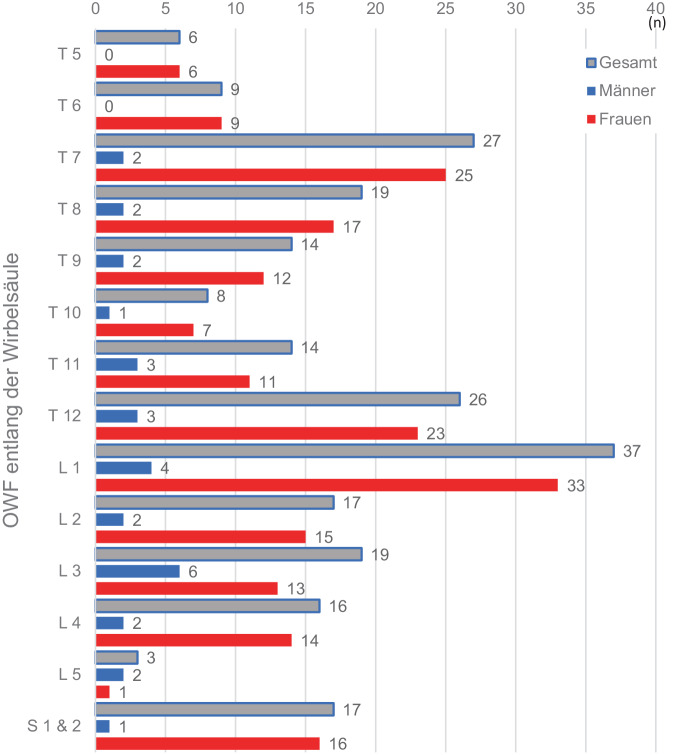
Distribution der osteoporotische Wirbelkörperfraktur (OWF) entlang der Wirbelsäule. Oberhalb von Brustwirbelkörper 5 finden sich keine Frakturen. Eine Häufung von OWF findet sich im mittleren Bereich der Brustwirbelsäule, thorakolumbal und sakral

Die Frakturdistribution entlang der Wirbelsäule ist in Abb. 4 dargestellt.

**Abb. 5:**
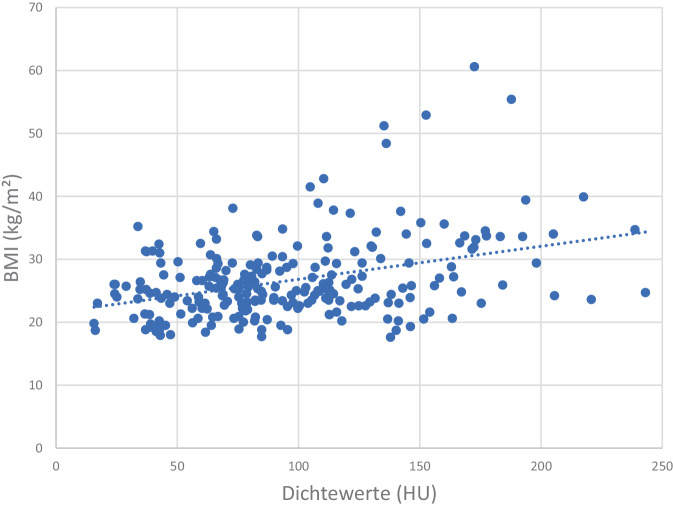
Abhängigkeit der HU-Werte von dem BMI. Bei Pat. mit niedrigem Body-Mass-Index (*BMI*) finden sich die niedrigsten Dichtewerte (HU)

**Abb. 6:**
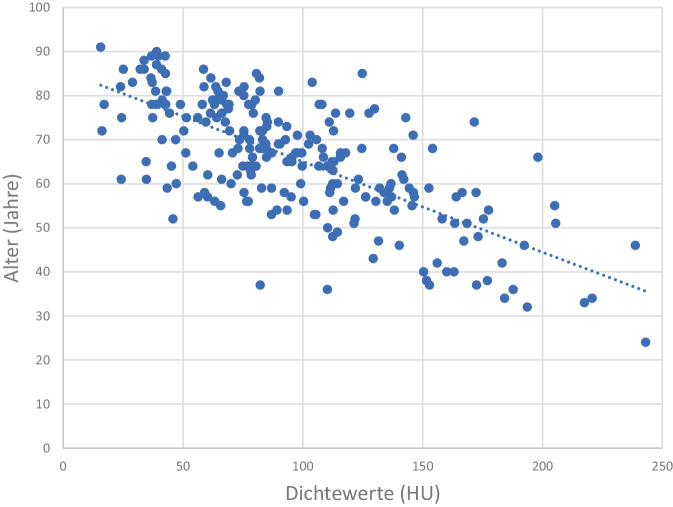
Abhängigkeit der HU-Werte von dem Pat.-Alter. Bei Pat. mit hohem Alter finden sich signifikant die niedrigsten Dichtewerte (HU)

Es fand sich eine direkte Korrelation zwischen Dichtewerten in HU und dem jeweiligen BMI, Pat. mit einem niedrigen BMI tendieren zu einer niedrigen Knochendichte (Abb. 5). Ein umgekehrtes Verhalten zeigte sich zwischen den HU-Dichtewerten und dem Pat.-Alter, hier zeigen Pat. mit hohem Alter signifikant (*p* < 0,001) die niedrigsten Dichtewerte (Abb. 6).

**Abb. 7:**
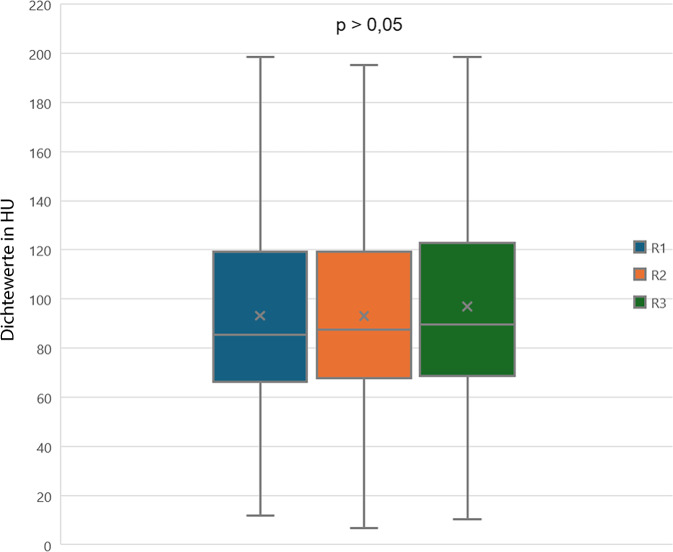
Bei der spongiösen Dichtebestimmung durch drei unabhängige Untersucher (*R*) ergibt sich kein signifikanter Unterschied

Unabhängig von den Untersuchern wurden vergleichbare HU-Werte bestimmt (*p* > 0,05), (Abb. 7).

**Abb. 8:**
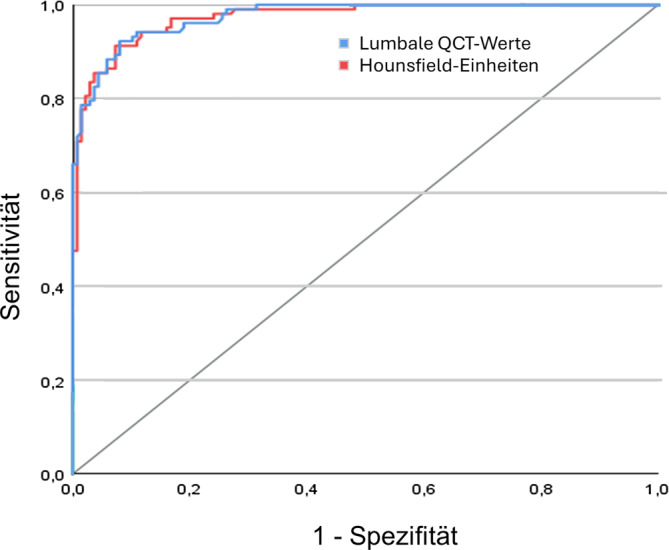
Die ROC(„receiver operating characteristic“)-Kurvenanalyse zeigt keinen Unterschied zwischen den AUC(„area under the curve“)-Hounsfield-Einheiten und der AUC-Lumbal-QCT (quantitative Computertomographie) (*p* = 0,395) für die Frakturrisikobestimmung. Ein Cut-off-Wert der Hounsfield-Einheiten (HU) zur optimalen Klassifizierung von Pat. mit und ohne Frakturen wurde anhand des maximalen Youden-Index bestimmt. Bei Patienten mit < = 70 HU wird von Frakturen ausgegangen (Sensitivität = 0,91; Spezifität = 0,93). Diese diagnostischen Maßnahmen sind mit denen der lumbalen QCT vergleichbar (Sensitivität = 0,92; Spezifität = 0,92; Cut-off < = 60 mg/cm^3^)

Mittels ROC-Kurvenanalyse lässt sich zeigen, dass die Aussagekraft für das Risiko für das Auftreten von OWF mittels HU-Werten sehr hoch ist und sich mit einem *p* = 0,395 kein signifikanter Unterschied zur QCT findet (Abb. 8).

## Diskussion

Aufgrund der hohen signifikanten Korrelation lassen sich die spongiösen Dichtemessungen in HU-Werten in quantitative KMG-Werte in mg/cm^3^ umrechnen, womit eine gute Abschätzung einer Demineralisation und Schwere der Osteoporose möglich wird. Hierzu passen auch die Untersuchungen von Buenger et al. [4] wobei an 369 Pat. mit einem ähnlichen Durchschnittsalter wie unsere Pat., sich eine hohe Korrelation zwischen den HU aus nativen CT-Schnittbildern und QCT-Werten fand. In einer In-vitro-Stude an 22 älteren Körperspendern kommen Schröder et al. [33] zu einer vergleichbaren Korrelation. Die unterschiedlichen Umrechnungsformeln aus den drei Untersuchungen sind in Tab. 5 aufgeführt, prozentuale Unterschiede der Umrechnungen von HU in QCT-Werte in mg/cm^3^ werden exemplarisch für 100 HU dargestellt. Eine Abweichung um 8,7 % bei Schröder et al. [33] nach unten, im Vergleich zum eigenen Kollektiv, ist wahrscheinlich bedingt durch einen vorhandenen Fettfehler [13], sodass es bei den älteren Körperspenden mit manifester Osteoporose zu einer Überschätzung der Demineralisation kam. Eine Abweichung um 9,6 % bei Buenger et al. [4] nach oben, im Vergleich zum eigenen Kollektiv, ist wahrscheinlich bedingt durch weniger schwere Osteoporosepatienten und damit vermutlich geringerem Fettfehler. Insgesamt zeigt sich hier ein gewisser Einfluss der Patientengruppe selbst auf die Steigung der Regressionsgrade und damit auf die jeweilige Umrechnungsformel von HU-Werten in QCT-Werte (mg/cm^3^). Wenn im Einzelfall die Diagnose mittels HU-Bestimmung nicht sicher möglich ist, könnte dieses durch eine zusätzliche Dual-energy QCT korrigiert werden [13, 15].

**Tab. 5:** Gegenüberstellung der eigenen Ergebnisse mit zwei methodisch vergleichbare Studien

Studiengruppe	DSA (in Jahren)	Umrechnungsformel von HU in QCT-Werte (mg/cm^3^)	Absolutwert und prozentuale Abweichung bei 100 HU im Vergleich zu den eigenen Ergebnissen
**Eigene Ergebnisse**	**64,9** **min.: 24; max.: 91**	**QCT-Wert** **=12,1** **+** **(0,68** **×** **HU)**	**80,1** **mg/cm**^**3**^
Buenger et al. 2021 [4]	66,98 min.: 28; max.: 92	QCT-Wert =17,8 + (0,7 × HU)	87,8 mg/cm^3^ *um 9,6* *% größer*
Schröder et al. 2023 [33]	81,1 min.: 66; max.: 102	QCT-Wert =13,7 + (0,6 × HU)	73,7 mg/cm^3^ *um 8,7* *% kleiner*

*DSA* Durchschnittsalter, *QCT* quantitative Computertomographie

Insgesamt zeigt sich bei einem niedrigen BMI (Abb. 5; [33]), einem zunehmenden Alter (Abb. 6; [18, 33]) und insbesondere steigender Frakturzahl ein signifikanter Abfall der Knochendichtewerte (Abb. 3a und b; [44]). Hinsichtlich einer Diskriminierung von Pat. mit und ohne OWF finden Zou et al. [44] zu uns vergleichbare Ergebnisse, bei einer spongiösen Dichte < 70 HU sind OWF wahrscheinlich. 70 HU ergeben nach Xq = 12,1 + (0,68 × HU) 59,7 mg/cm^3^, welches als Frakturschwelle in guter Übereinstimmung zu einer früheren QCT-Auswertung steht, wo 60 mg/cm^3^ angegeben werden [1]. Des Weiteren entsprechen 100 HU 80,1 mg/cm^3^, welches gut zum festgelegten quantitativen Osteoporoseschwellenwert von 80 mg/cm^3^ passt [9, 11, 13]. Um mögliche Dichteschwankungen einzelner Wirbelkörper auszugleichen, sehen, wie von uns durchgeführt, auch Scheyerer et al. [28] die Messung an mindestens drei verschiedenen Lendenwirbelkörpern für wichtig an.

Grundsätzlich eignet sich die spongiöse Dichtebestimmung am gesamten Achsenskelett, wobei für die zervikalen, thorakalen, lumbalen und sakralen Bereiche unterschiedliche Schwellenwerte zu berücksichtigen sind [3, 30, 39]. In kraniokaudaler Richtung zeigt sich hierbei eine Abnahme der Dichtewerte entlang der Wirbelsäule ohne oder mit Osteoporose [3, 30, 32, 34, 39]. Die Frakturdistribution unserer Pat. (Abb. 4) zeigt eine vergleichbare Verteilung zu anderen In-vivo- und In-vitro-Untersuchungen [3, 23, 30]. Die niedrigsten spongiösen Dichtewerte als Ausdruck einer schweren Osteoporose finden sich in der LWS bei gleichzeitig vorhandenen Sakruminsuffizienzfrakturen, andererseits sind diese Frakturen allein ein deutlicher Indikator für Osteoporose [27].

Erfahrene Untersucher kommen bei den HU-Messungen zu einer hohen Inter-Rater-Reliabilität [33, 38], auch in der vorliegenden Studie kam es zu keinem signifikanten (*p* > 0,005) Unterschied zwischen den drei Radiologen (Abb. 7).

### Weitere klinische Bedeutung der HU-Dichtebestimmung

Choi et al. [6] finden bei DEXA-Messungen mit degenerativen Wirbelsäulenveränderungen tendenziell zu hohe Dichtewerte, wobei die Dichtebestimmung mittels HU eher die tatsächliche Situation widerspiegelt. CT-HU-Werte können hier als ergänzende Methode, der mittels DEXA nicht diagnostizierten spinalen Osteoporose korrigieren [43]. Für die Risikoabschätzung von mechanischen Komplikationen nach einer Wirbelsäulendeformitätsoperation bei Erwachsenen können HU-Schwellenwerte hilfreich sein [5, 41]. Die Bestimmung von HU in der Wirbelsäule hat auch Einfluss auf die Beurteilung von Komplikationen nach Wirbelsäulenoperationen, so lassen sich Schraubenlockerungen bei niedrigen HU-Werten besser als mit anderen Methoden vorhersagen [20]. Bei Zustand nach einer perkutanen Ballonkyphoplastie zur Versorgung von OWF sind niedrige HU-Werte ein Prädiktor für Anschlussfrakturen [40]. Ähnliches findet sich nach kurzstreckiger lumbaler Spondylodese, wobei HU-Werte < 80 im ventralen Drittel benachbarter Wirbel ein Risiko für Folgefrakturen darstellen [24]. Auch der Anstieg der Knochendichte nach osteoanaboler Therapie kann mittels HU-Messungen im Verlauf zuverlässig gemonitort werden [8].

### Stärken und Limitationen

Als Stärke der Studie lassen das weite Spektrum des Pat.-Alters, der BMI-Werte und das Vorhandensein von Pat. ohne und mit manifester Osteoporose eine große Range von HU-Werten und damit sichere Korrelationen erwarten.

Um in unterschiedlichen Abteilungen vergleichbare Werte zu bekommen, sollten die HU-Bestimmungen unter standardisierten Bedingungen erfolgen.

Das Patientenkollektiv hinsichtlich Pat.-Alter, Ausmaß der Osteoporose, BMI und Geschlecht haben wahrscheinlich einen Einfluss auf die Regressionsgrade und Umrechnungsformel, welches im Vergleich mit anderen Studien zu bedenken wäre (Tab. 2). Bei alten Pat. mit einer schweren, manifesten Osteoporose hat die mögliche Verfettung des trabekulären Raums einen Einfluss hinsichtlich der Überschätzung der Schwere der Demineralisation.

Es liegt kein Vergleich mit DEXA-Untersuchungen vor.

Die Ergebnisse aus unserer retrospektiven Studie sollten nach Möglichkeit durch eine prospektive randomisierte Multicenter-Folgeuntersuchung untermauert werden.

## Fazit für die Praxis

- Unter Berücksichtigung der gewonnenen Ergebnisse erscheint eine opportunistische Auswertung allein mittels Hounsfield-Unit-Werten im nativen CT der Lendenwirbelsäule (LWS) zur Abschätzung einer Osteoporose und Bestimmung des Risikos für osteoporotische Wirbelkörperfraktur, auch durch unterschiedliche Untersucher, valide durchführbar.
- Eine zusätzliche QCT/Dual-energy QCT oder DEXA der LWS kann in Grenzfällen die definitive Diagnose klären, erscheint jedoch bei vorliegender CT in den meisten Fällen nicht notwendig, eine weitere Strahlenbelastung sowie zusätzliche Kosten entfallen.

## Data Availability

Die erhobenen Datensätze können auf begründete Anfrage in anonymisierter Form beim korrespondierenden Autor angefordert werden. Die Daten befinden sich auf einem Datenspeicher am Westküstenklinikum Heide, Deutschland.

## Abkürzungen

AUC: „Area under the curve“
BMD: „Bone mineral density“
BMI: Body-Mass-Index
BWS: Brustwirbelsäule
DEXA: Dual-Energie-Röntgenabsorptiometrie
DSA: Durchschnittsalter
GE: General Electric
HU: Hounsfield-Unit/Hounsfield-Einheit
KMG: Knochenmineralgehalt
LWK: Lendenwirbelkörper
LWS: Lendenwirbelsäule
M: Mittelwert
n: Anzahl
OVF: „Osteoporotic vertebral fracture“
OWF: Osteoporotische Wirbelkörperfraktur
Pat (Pt): PatientInnen
QCT: Quantitative Computertomographie
Q1–Q3: mit erstem und drittem Quartil
ROC: „Receiver operating characteristic“
ROI: „Region of interest“
SD: Standardabweichung
STIR: „Short-tau-inversion-recovery“
WS: Wirbelsäule
Xq: QCT-Wert in mg/cm^3^
